# Supplementary material for: Regulatory T Cells with Additional COX-2 Expression Are Independent Negative Prognosticators for Vulvar Cancer Patients
Source: Int J Mol Sci. 2022 Apr 22;23(9):4662. doi: 10.3390/ijms23094662 (PMC9099805; doi:10.3390/ijms23094662)
Supplement: Supplementary file 1 [file ijms-23-04662-s001.zip › ijms-1601769-supplementary.pdf]

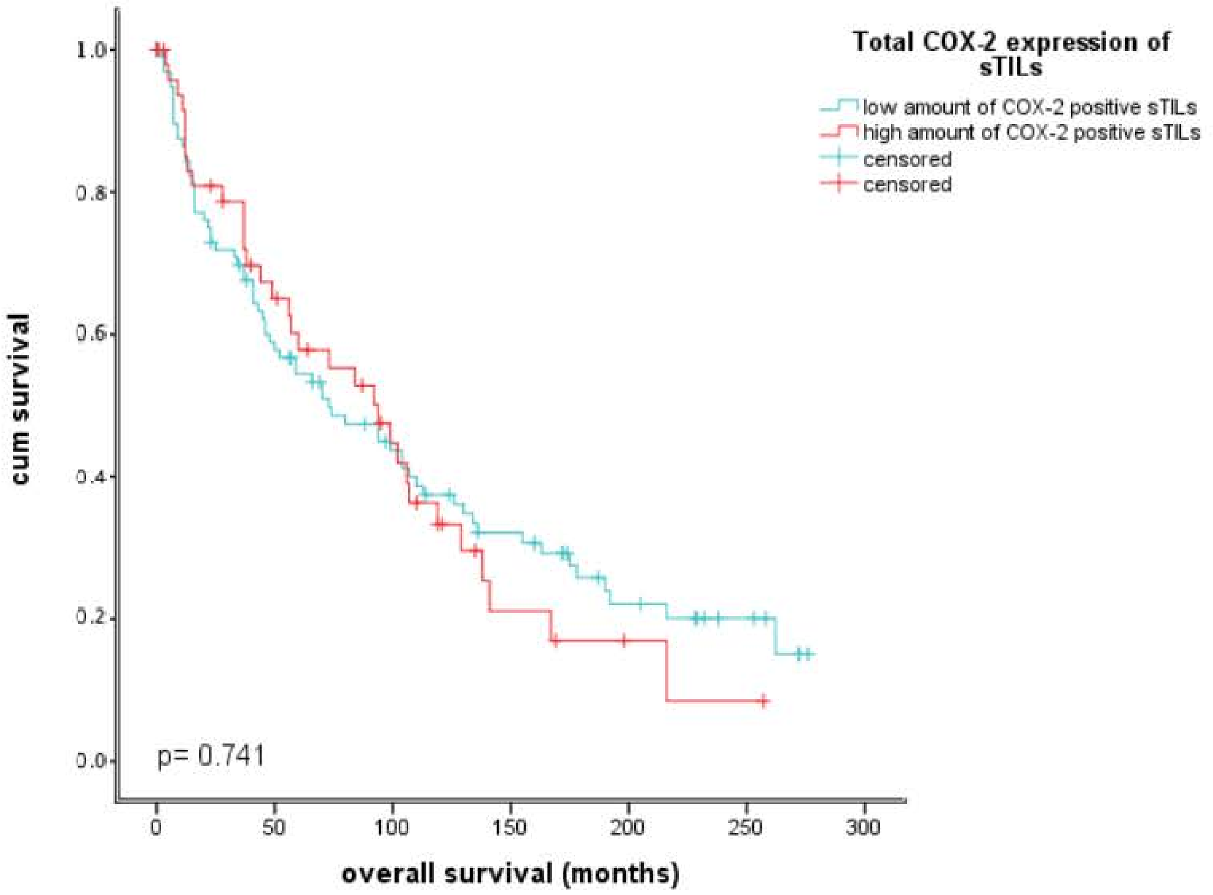

Supplementary Figure S1. Analysis of total COX-2 expression of sTILs in vulvar carcinoma cases concerning overall survival. The red line shows the high proportion of COX-2 positive sTILs, whereas the blue line illustrates the low amount of COX-2 positive sTILs in vulvar carcinoma. Despite the lack of significance ($p = 0.741$), patients with high amount of COX-2 positive sTILs appear to have a lower survival rate after 120 months.

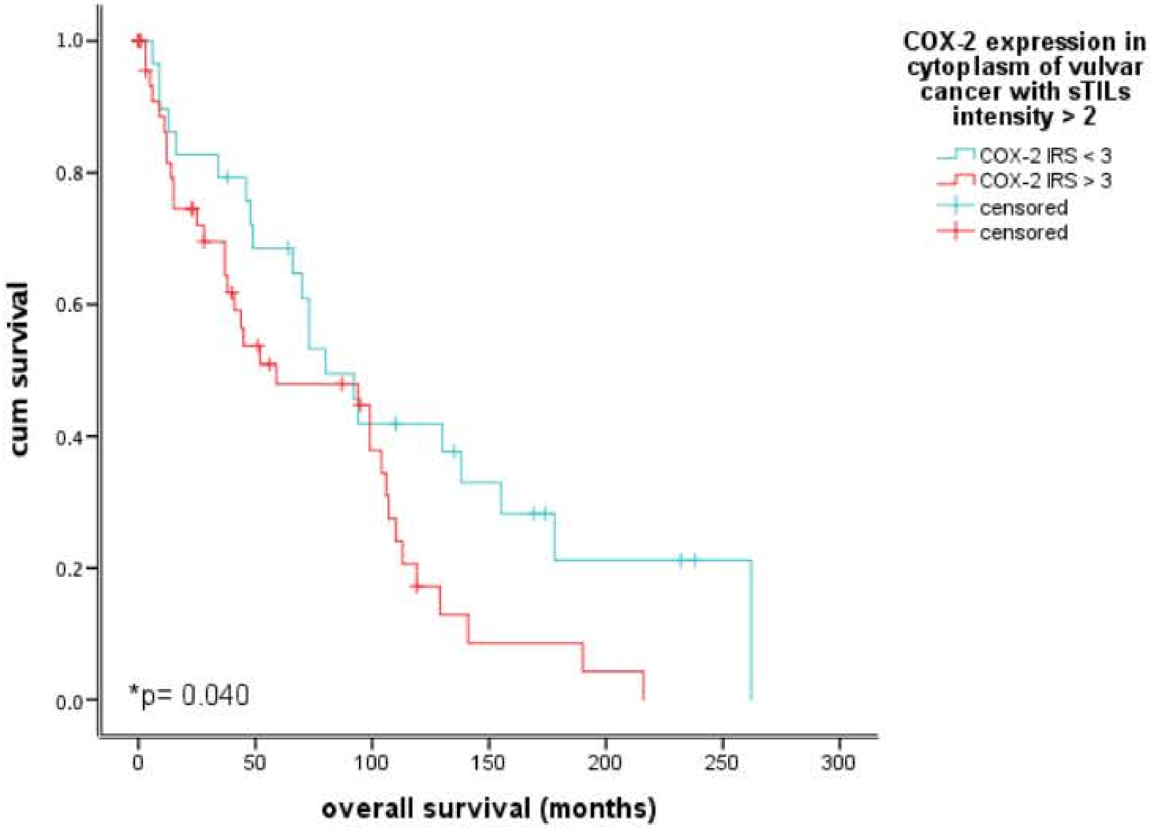

Supplementary Figure S2. Analysis of the impact on overall survival of COX-2 expression in vulvar cancer among cases with sTILs of high COX-2 intensity >2. The red line shows the cases among the high intensity sTILs with high COX-2 expression of IRS >3 in tumor tissue; the blue line shows those with low COX-2 expression of IRS <3 in tumor tissue. There is a significant survival advantage of patients with a COX-2 expression of IRS>3 in carcinoma tissue with sTILs intensity >2 (*p= 0.040).

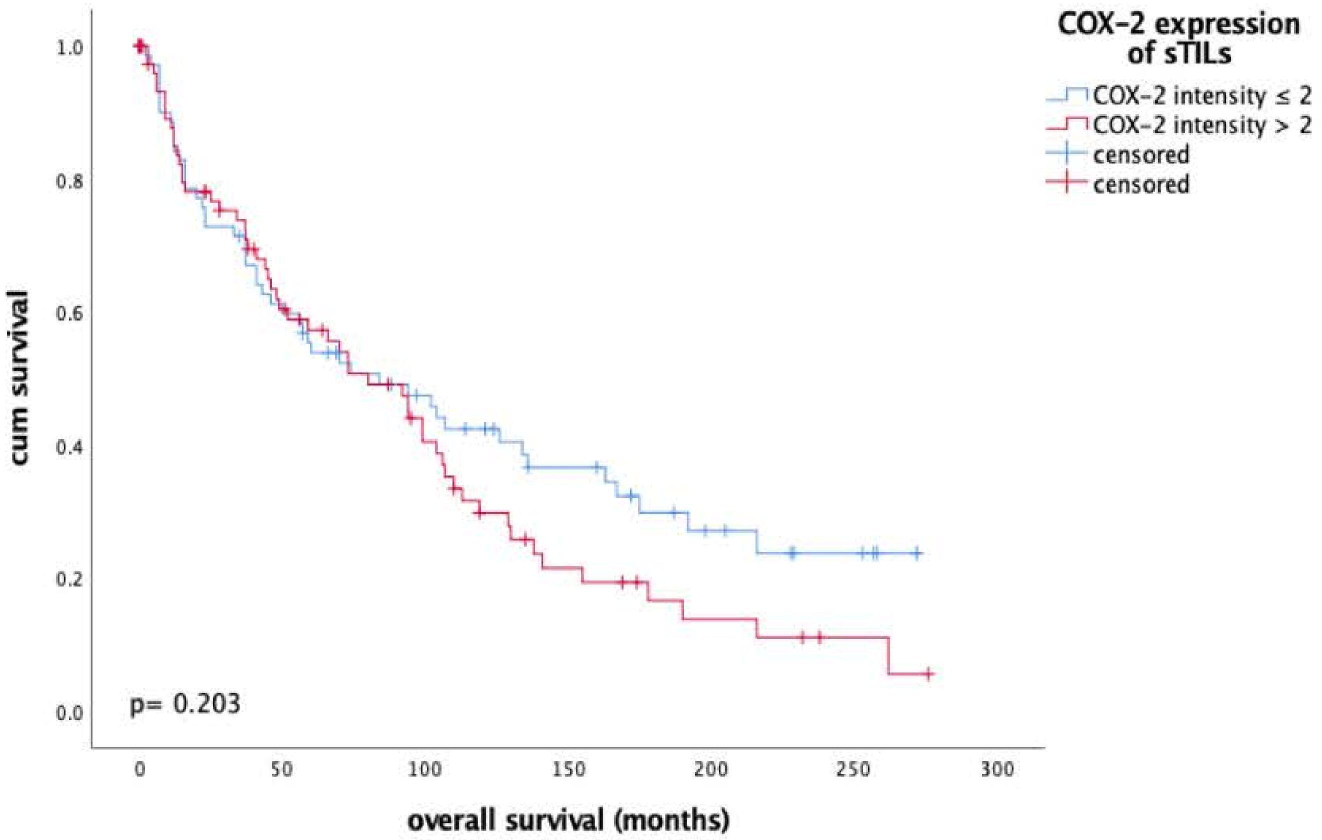

Supplementary Figure S3. The Kaplan- Meier curve shows a clear trend of an overall survival advantage of those patients who have weaker COX-2 expression of intensity ≤ 2 (blue line) of sTILs than those who have stronger COX-2 expression of intensity > 2 (red line) (p= 0.203).

4a

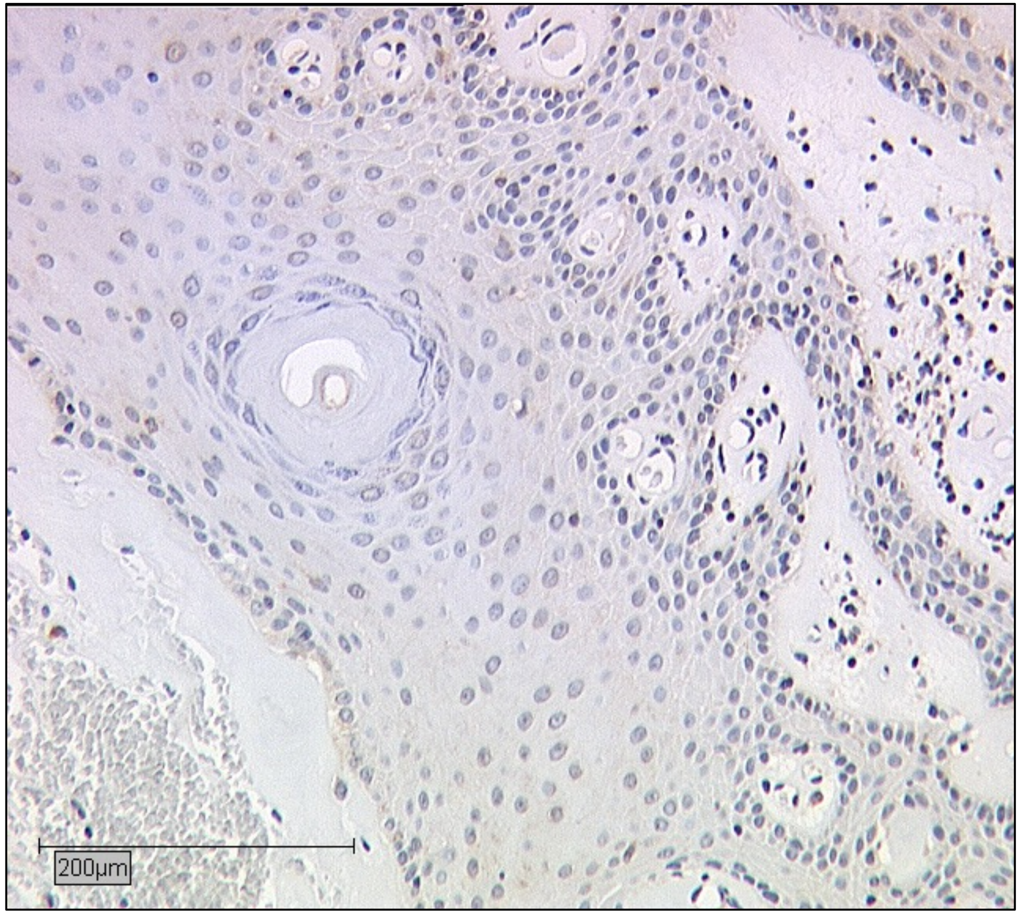

4b

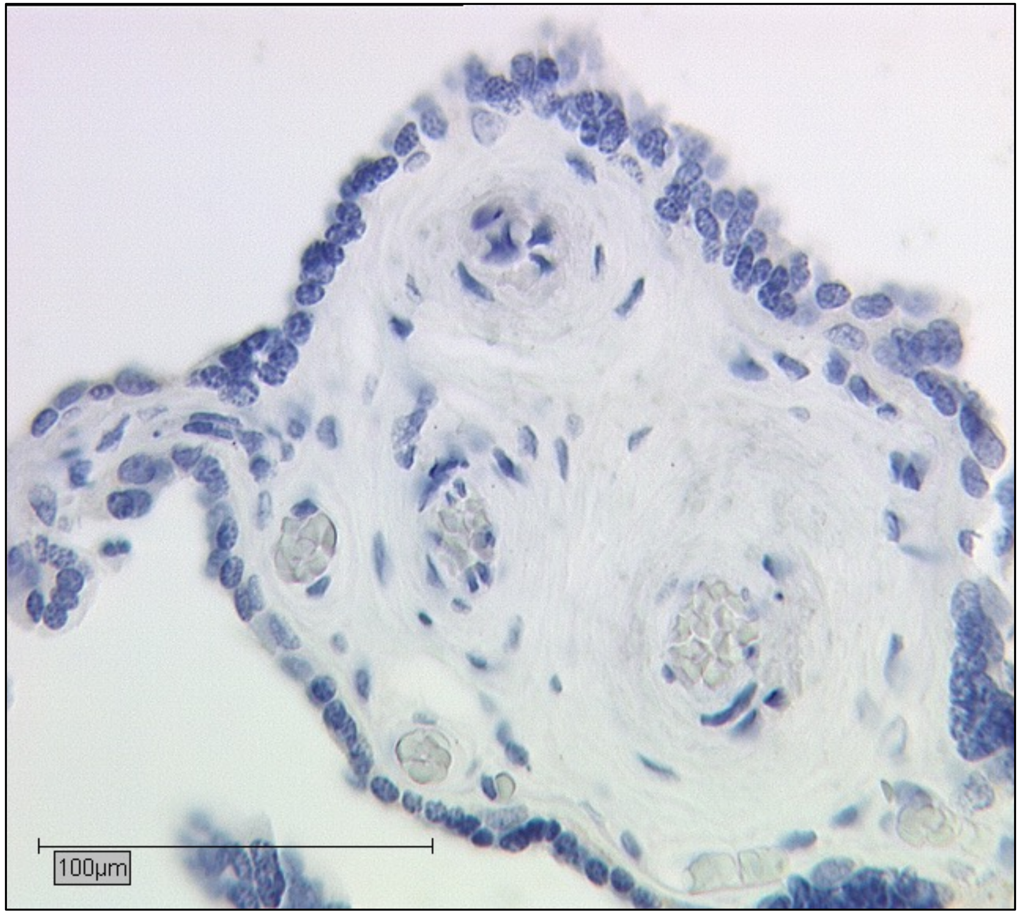

Supplementary Figure S4. Figure 4a shows COX-2 immunohistochemistry staining on benign vulvar tissue (10x magnification). Figure 4b shows a negative staining control of COX-2, here using placental tissue as an example (25x magnification).

5a 5b

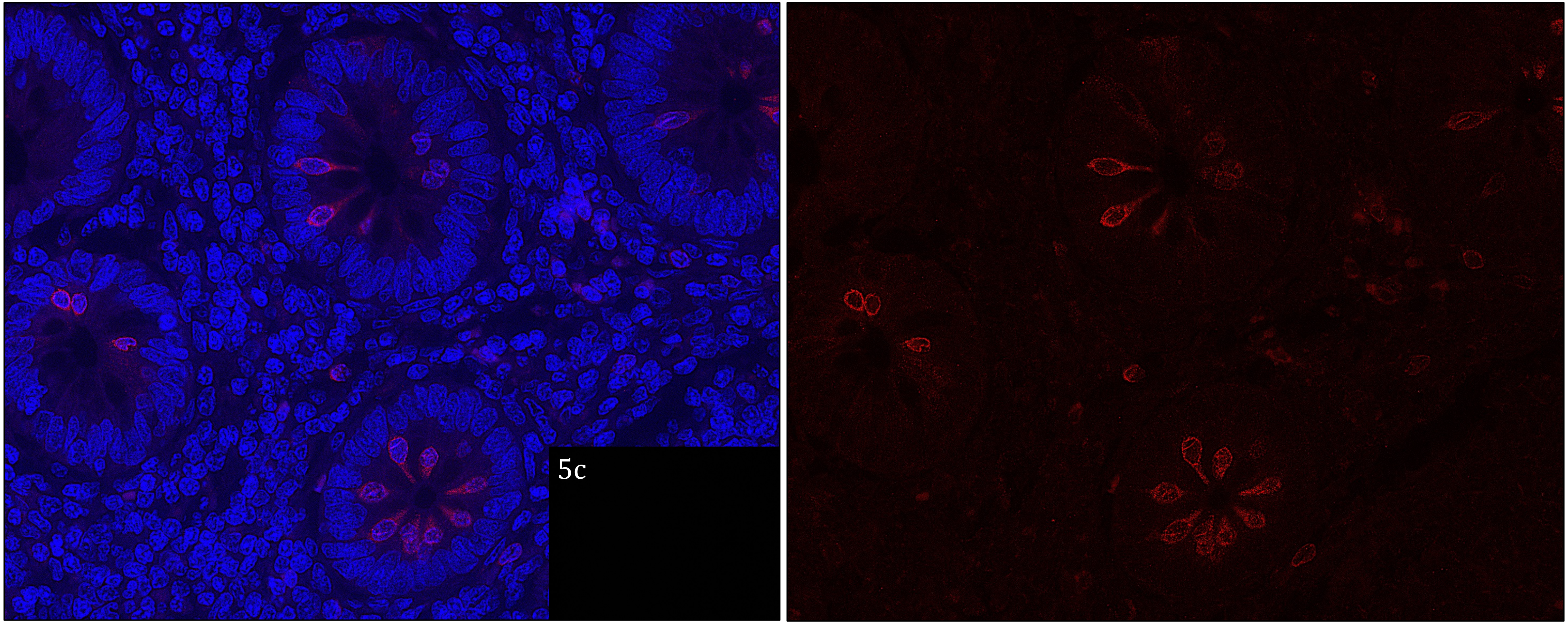

Supplementary Figure S5. Figure 5a show immunofluorescence staining as a positive control for COX-2 (red, Figure 5b) in cytoplasm using appendiceal tissue as an example (magnification 40x). The insert Figure 5c shows negativ control staining of rabbit second-antibody (used for FOXP3, CD56, CD68 with Cy-2 for green staining) with no unspecific staining.

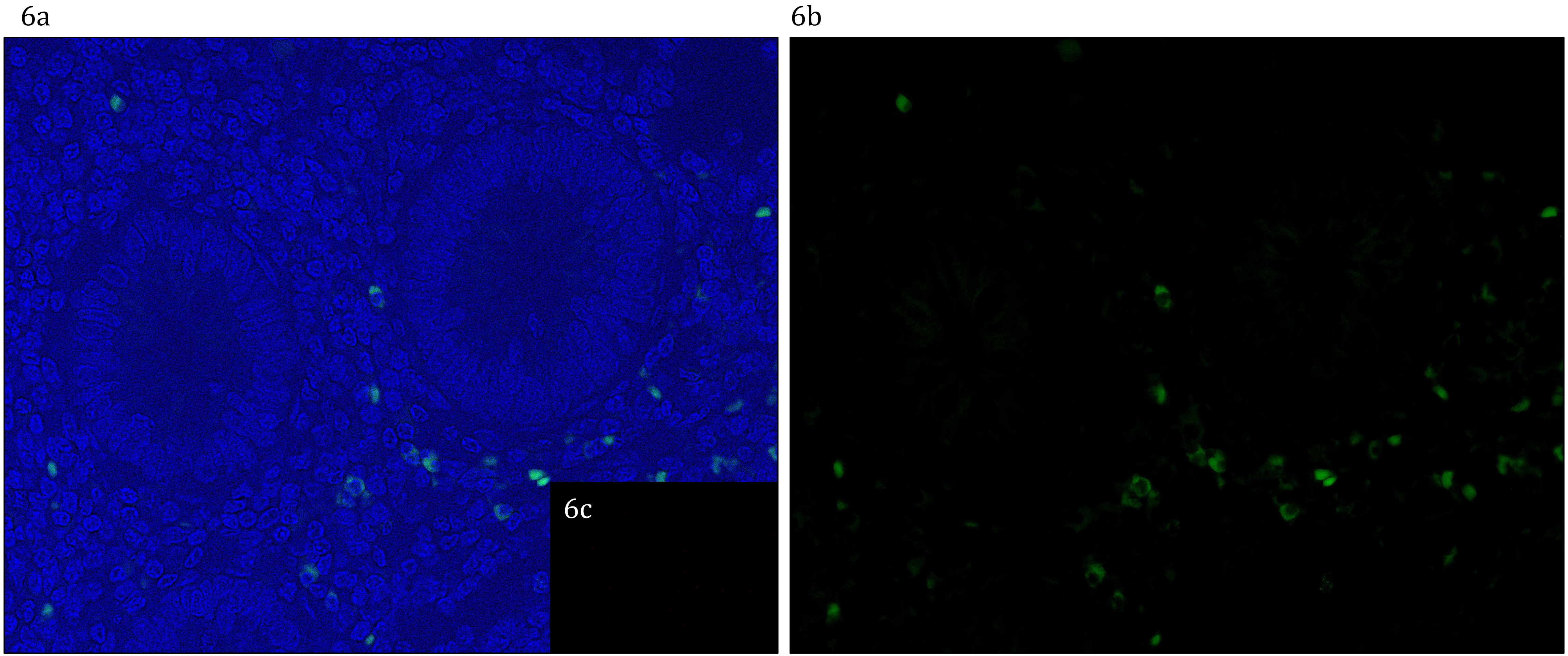

Supplementary Figure S6. Figure 6a show immunofluorescence staining as a positive control for FOXP3 (green, Figure 6b) using appendiceal tissue as an example (magnification 40x). The Figure shows nuclear staining of FOXP3, but also in few cases cytoplasmic staining, which is in accordance to human protein atlas showing also partial cytoplasmic staining of FOXP3. The insert Figure 6c shows negativ control staining of mouse second-antibody (used for COX-2 with Cy-3 for red staining) with no unspecific staining.
